# Daily Dispatch

**Published:** 1879-06-20

**Authors:** 


### *THE DAILY DISPATCH.*

This is a new daily paper recently started in Atlanta, and we cheerfully recommend it to our readers, who may desire to take an Atlanta paper. It is published by Miller & Dickson, experienced newspaper men, at $6.00 per annum or $3.00 for 6 months. The paper is presided over by John H. Martin, late of the *Columbus Times*, an able and experienced editor. The Dispatch gives promise of permanency and usefulness. Those connected with it are courteous and high-toned gentlemen, and the public will doubtless heartily welcome and encourage this enterprise.

